# Current Requirements and Attitudes Toward Research: A Survey-Based Analysis of Orthopedic Surgery Programs

**DOI:** 10.7759/cureus.32570

**Published:** 2022-12-15

**Authors:** Ameen Barghi, Eric Gruenberger, Rachel Gottlieb, Kiera Lunn, Kyle D Paul, Reily Cannon, Brent A Ponce, George S. M. Dyer, James Herndon

**Affiliations:** 1 Orthopedics, Atrium Health Wake Forest Baptist Health, Winston-Salem, USA; 2 Orthopedics, Hughston Foundation, Columbus, USA; 3 Medicine, University of Michigan, Ann Arbor, USA; 4 Orthopedic Surgery, Massachusetts General Hospital, Boston, USA; 5 Orthopedics, University of Alabama School of Medicine, Birmingham, USA; 6 Medicine, Touro College of Osteopathic Medicine, Henderson, USA; 7 Orthopedic Surgery, Hughston Clinic, Columbus, USA; 8 Orthopedic Surgery, Harvard Medical School, Boston, USA

**Keywords:** orthopedics, academic residency, acgme, research requirements, orthopedic surgery

## Abstract

Objective

The Accreditation Council for Graduate Medical Education (ACGME) guidelines require scholarly activity but do not specify what research-related activity is necessary to meet this requirement. The current components and opinions regarding research and its implementation that qualify as scholarly activity are unknown among US orthopedic surgery programs. We aimed to survey program directors of orthopedic surgery programs to evaluate and better understand the current state of research during training.

Design

A survey was sent to the program directors of all ACGME-accredited orthopedic surgery between 2019 and 2020 with questions evaluating each program’s research requirements and barriers to improvement.

Results

One-hundred eighteen (N=118) surveys were collected from 94 academic (79.6%) and 24 community (21.4%) programs. Although nearly all (97.5%) programs required research for graduation, only 45% of them allotted protected time: 52 academic programs (55.3%) allotted a median of nine weeks (interquartile range (IQR): 8-12 weeks) of dedicated time and 13 community programs (54.2%) allotted six weeks (IQR: 4-28 weeks) (p=0.595). We distinguished dedicated research as either consecutive weeks or a formal research track for a year. All programs indicated a desire for an increased focus on basic science compared to the current focus on outcomes-based research (p=0.04). The greatest identified obstacle to research improvement reported by community programs was faculty and resource commitment (p=0.003). The overall level of satisfaction with the current research experience among directors is 50.8%.

Conclusion

Despite differences between academic and community programs, directors agree on shifting the focus of research toward basic science. To improve preclinical research, additional time may be required, and individualized improvement plans should be undertaken at academic and community programs alike.

## Introduction

With a shift to evidence-based medicine over the past 30 years, the availability of high-quality research in orthopedics and the skills to interpret and utilize research are increasingly important for the delivery of care and improved patient outcomes [1]. The Accreditation Council for Graduate Medical Education (ACGME) sets forth core competencies for orthopedic residents, which include an expectation for residents to effectively assess the available literature for evidence to inform and improve practice [2]. Furthermore, the ACGME requires scholarly activity for trainees, including orthopedic residents and orthopedic fellows. There is no specific mandate for what qualifies as scholarly activity, but many surgical specialties stipulate research activities to fulfill this requirement [1,3,4]. Despite this, many orthopedic residencies may not have protected research times [5].

At present, there is little evidence of what orthopedic residencies require for the fulfillment of the scholarly activity requirement, the current state of research at orthopedic residency programs, or the perceived barriers to meeting the same academic standards as other surgical specialties. In this study, we aimed to better understand how orthopedic residency programs implement research into training and the program directors’ perceptions of the value of research during training. We conducted a survey of program directors at ACGME-accredited orthopedic residencies and fellowships to better understand the current state of research during training.

## Materials and methods

Methods

With Institutional Review Board (IRB) approval (2019P001219), the authors developed a survey using RedCap® to determine orthopedic surgery programs (n=74) and fellowship directors’ (n=44) demographics, program type (academic versus community), and whether research was required for graduation. For programs requiring research, respondents indicated the components, timing, opinions, perceived barriers, and satisfaction levels for the current research experience. Surveys were emailed to all ACGME-accredited orthopedic residency and fellowship programs between 2019 and 2020, and responses were collected over a four-month period bridging the 2019 and 2020 academic years. Our study timeline was not impacted by the COVID-19 pandemic, as our collected data had been finalized before the start of the 2020 academic year.

Statistics

Significance and beta values were set at 0.05 and 0.2, respectively. We assessed power using the Student’s t-test and the Shapiro-Wilk test to assess the normality of distribution. Descriptive statistics are reported as mean ± standard deviation (SD) for parametric data and median (interquartile range (IQR)) for nonparametric data. Categorical data were assessed with chi-squared analysis and Mann-Whitney U test for nonparametric data; nonparametric continuous data were analyzed using the Kruskal-Wallis test, and Friedman’s two-way analysis of variance (ANOVA) was applied to related samples. Likert scales were used to assess subjective data. The Statistical Package for the Social Sciences (SPSS) version 27 (IBM SPSS Statistics, Armonk, NY, USA) was used for statistical analysis.

## Results

A total of 118 surveys were completed. Academic programs, as defined by program/fellowship directors, accounted for 79.6% of responses (N=94), and community programs accounted for 21.4% of responses (N=24). Forty-four (37.3%) of the respondents were fellowship directors, 39 (88.6%) of whom were in academic programs. Formal research tracks were offered at six academic programs and one community program.

Academic programs were four times more likely to receive public funding compared to community programs (p=0.044) (Table 1). Institutional or departmental grants for the primary purpose of funding research at the program were reported by 57.1% and 16.7% of public and private institutions, respectively (p=0.010). Additional demographic information for program directors and their programs is summarized in Table 1.

**Table 1:** Characteristics of academic and community programs and directors (N=118) Results are based on nonempty rows and columns in each innermost subtable. *Pearson’s chi-squared statistic is significant at the p=0.05 level. LRs are reported if a significant difference is identified, within the parentheses. ^a^More than 20% of cells in this subtable have expected cell counts of less than 5. Chi-squared test results may be invalid. ^b^The minimum expected cell count in this subtable is less than one. Chi-squared test results may be invalid. ^c^Levene’s test for equal variance was not significant at p=0.05 assuming equal variances, indicating that variances were not equal (F statistic=0.085, p=0.771). LR: likelihood ratios, IQR: interquartile range

		Academic			Community			p-value (LR)
		Count	%	Median (IQR)	Count	%	Median (IQR)	
Gender	Non-binary	1	1.1%		1	4.2%		0.513^a,b^
	Male	77	81.9%		20	83.3%		
	Female	16	17%		3	12.5%		
Year as program director	First to second	20	21.3%		6	25%		0.717
	Third to fifth	20	21.3%		3	12.5%		
	Sixth to 10th	24	25.5%		8	33.3%		
	11th or more	30	31.9%		7	29.2%		
Institution	Private	49	52.1%		18	75%		0.044^*^ (4.3)
	Public	45	47.9%		6	25%		
Location	Rural	7	7.4%		5	20.8%		0.008^*^ (8.8)
	Suburban	14	14.9%		8	33.3%		
	Urban	73	77.7%		11	45.8%		
Research required for graduation		91	96.8%		24	100%		0.375^a,b^
Graduating class size				5 (3-6)			3 (3-5)	0.946^c^
% of graduates not completing research requirement	Always complete	21	72.4%		4	44.4%		0.062^a,b^
	<5%	8	27.6%		3	33.3%		
	6%-25%							
	26%-50%				1	11.1%		
	>50%				1	11.1%		

Research requirements and components were recorded and compared. Three (academic) programs did not require research for graduation; of the 115 (97.5%) remaining programs that required research, only 65 (56.5%) provided dedicated time for research. Fifty-two (55.3%) academic programs allotted a median of nine weeks (IQR: 8-12 weeks) of dedicated time, while 13 (54.2%) community programs allotted six weeks (IQR: 4-28 weeks) (p=0.595). While we found that only 96.8% of academic programs require research for graduation as compared to 100% of community programs requiring research for graduation, this result was not significant. Most programs provided research training for their residents in the form of two or three annual lectures, and most projects were original ideas (versus assigned topics) at both academic and community programs. Research oversight was similar among all programs (p=0.131); program directors typically provided general oversight, and individual projects were universally assigned at least one faculty mentor for guidance and accountability. These results are displayed in Table 2.

**Table 2:** Research components Note: Bolded values are significantly different at p<0.05 in the two-sided test of equality for column proportions (N=65, U statistic=306.00, p=0.595). This excluded programs with a one-year dedicated research track. Tests are adjusted for all pairwise comparisons within a row of each innermost subtable using the Bonferroni correction. ^a^More than 20% of cells in this subtable have expected cell counts of less than 5. Chi-squared test results may be invalid. ^b^The minimum expected cell count in this subtable is less than one. Chi-squared test results may be invalid. ^1^Tests were not done because the column proportions are equal to one or zero IQR: interquartile range, PGY: postgraduate year

		Academic			Community			p-value
		Number of programs	%	Median (IQR)	Number of programs	%	Median (IQR)	
Completed research product required for graduation		75^a^	63.6%		19^a^	16.1%		0.946^a^
Formal track year offered		6^a^	15.8%		1^a^	2.6%		0.517^a^
Dedicated research period provided (weeks)		52^a^	55.3%	9 (8-12)	13^a^	54.2%	6 (4-28)	0.846^a^
Accepted research products	Manuscript	77^a^	83.7%		24^1^	100%		0.001^a,b*^
	Poster	16^a^	17.4%		13^b^	54.2%		
	Podium	28^a^	30.4%		11^a^	45.8%		
	Grand rounds	5^a^	5.4%		1^a^	4.2%		
	IRB submission or clinical trial	0^1^	0%		1^a^	4.2%		
Year during which dedicated research is performed	PGY1	1^a^	1.3%		3^b^	13.6%		0.000^a,b,*^
	PGY2	9^a^	11.4%		7^b^	31.8%		
	PGY3	13^a^	16.5%		6^a^	27.3%		
	PGY4	10^a^	12.7%		6^a^	27.3%		
	PGY5	3^a^	3.8%		5^b^	22.7%		
	Flexible timing	53^a^	67.1%		12^a^	54.5%		

Opinions on research

The only statistically significant difference of opinion between academic and community program directors was the rating of “neutral” regarding whether research is an integral part of orthopedic training (5.3% academic versus 20.8% community, p=0.03) (Table 3).

**Table 3:** Opinions on research Note: Values in the same row and subtable not sharing the same subscript are significantly different at p<0.05 in the two-sided test of equality for column proportions. Cells with no subscript are not included in the test. Tests assume equal variances. Results are based on nonempty rows and columns in each innermost subtable*. The chi-squared statistic is significant at the 0.05 level. Tests are adjusted for all pairwise comparisons within a row of each innermost subtable using the Bonferroni correction. ^a^More than 20% of cells in this subtable have expected cell counts of less than 5. Chi-squared test results may be invalid. ^b^The minimum expected cell count in this subtable is less than one. Chi-squared test results may be invalid. ^c^Levene’s test for equal variance was not significant at p=0.05 assuming equal variances, indicating that variances were not equal (F statistic=0.085, p=0.771). ^1^Tests were not done because the column proportions are equal to one or zero.

		Academic		Community		
		Count	% group	Count	% group	p-value
Research is an integral part of orthopedic surgery training	Disagree strongly	0^1^	0%	0^1^	0%	0.030^*,b^
	Disagree	0^1^	0%	0^1^	0%	
	Neutral	5^a^	5.3%	5^b^	20.8%	
	Agree	45^a^	47.9%	7^a^	29.2%	
	Agree strongly	44^a^	46.8%	12^a^	50%	
Research is an important part of a physician’s professional development	Disagree strongly	0^1^	0%	0^1^	0%	0.133^b,c^
	Disagree	2^a^	2.1%	1^a^	4.2%	
	Neutral	8^a^	8.5%	5^a^	20.8%	
	Agree	54^a^	57.4%	8^b^	33.3%	
	Agree strongly	30^a^	31.9%	10^a^	41.7%	
Rigor of research program is why trainees apply here	Disagree strongly	9^a^	9.6%	3^a^	12.5%	0.302^b,c^
	Disagree	27^a^	28.7%	10^a^	41.7%	
	Neutral	33^a^	35.1%	3^b^	12.5%	
	Agree	23^a^	24.5%	7^a^	29.2%	
	Agree strongly	2^a^	2.1%	1^a^	4.2%	

Research focus

**Figure 1:**
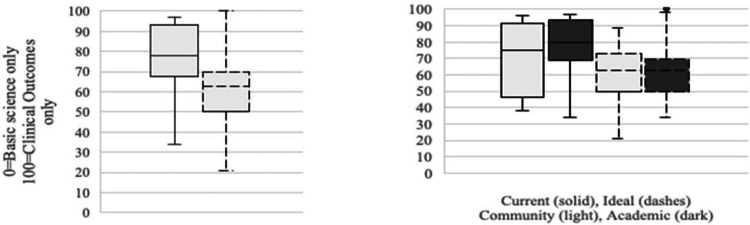
Opinions of all program directors on the current versus ideal focus of trainee research topics (left); academic and community program directors shared similar opinions (right) Left: Overall difference between the current (solid line) and ideal (dashes) research focus (Friedman’s two-way ANOVA statistic=4.235, p=0.040). Right: Current and ideal research focus of academic (dark box) and community (light box) programs compared (H=0.861, p=0.353) (N=118 respondents; H=0.041, p=0.840). ANOVA: analysis of variance

Program directors agreed on shifting the focus of research toward basic science using a sliding scale where “0” indicated “entirely basic science” and “100” indicated “entirely clinical or outcomes-based.” Overall, directors rated the current research focus at an average of 77.7 and an ideal focus of 62.5, suggesting more basic science-based research is desired (Friedman’s two-way 100 analysis of variance (ANOVA), p<0.04). As shown in Figure 1, no differences were found when stratifying responses by program type.

Barriers to research

**Figure 2:**
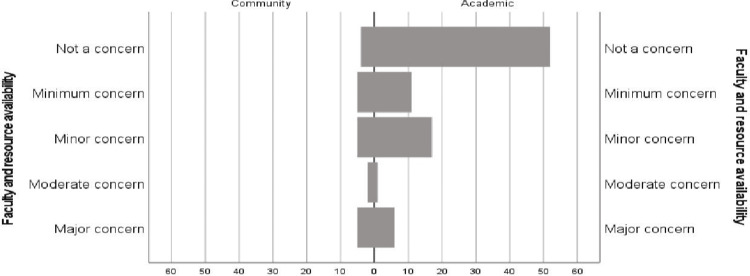
Level of concern over faculty and resource availability

Academic and community program directors reported a similar distribution of concerns over trainee perceptions of research, historical precedents, and attitudinal barriers to improving research at their individual programs. However, academic programs reported significantly lower concern over faculty and resource availability (59.8% versus 19%, Mann-Whitney U test, p=0.003) (Figure 2).

Satisfaction among trainees and faculty

**Figure 3:**
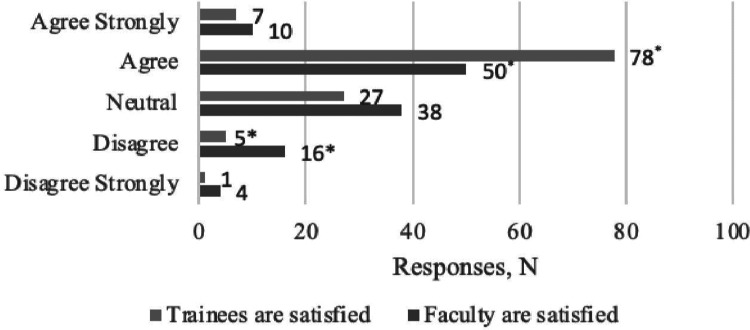
Trainee (light bars) versus faculty (dark bars) satisfaction at each program Forty-five (45) programs had both residents and directors report “agree” for overall satisfaction. *Significant difference between values

Pairwise comparison of trainee and faculty satisfaction levels as reported by the program directors, independent of program type, showed a higher proportion of dissatisfaction among faculty members (13.6% versus 4.2%), while trainees had proportionally higher satisfaction (66.1% versus 42.2%, p=0.003). Evaluated as an average score, however, no difference was found in the overall level of satisfaction of trainees versus faculty (p=0.296). Sixty-eight (57.6%) programs reported concordant satisfaction scores for faculty and trainees. Of these, 45 programs (overall: 38%) were satisfied with the current research experience (Spearman’s rho=0.556, p<0.001). No variables were found to be associated with higher concordance or satisfaction ratings. These results are summarized in Figure 3.

Fellowship directors

Forty-four (37.3%) respondents were fellowship directors, 39 (88.6%) of whom were in academic programs. Using multivariate regression, we found that fellowship director responses were similar to the residency program director responses for all variables after controlling for program type. Community fellowship directors reported a higher degree of faculty satisfaction with the current research experience (3.7 versus 3.2 out of 5.0, p<0.05) and a higher degree of concern over faculty and resource commitment to research (3.1 versus 2.0 out of 5.0, p<0.001). Fellowship directors also reported the desired shift toward basic science research with no difference between academic and community fellowships (p>0.5). Table 4 summarizes the results for variables found to be significantly different among those assessed for residency versus fellowship directors.

**Table 4:** Tests of between-subjects effects showing that program type (academic versus community), but not director type (residency versus fellowship), was associated with the observed differences between faculty satisfaction and concern over faculty and resource commitment Note: Bolded values are significantly different at p<0.05. ^a^More than 20% of cells in this subtable have expected cell counts of less than 5. Chi-squared test results may be invalid. ^b^The minimum expected cell count in this subtable is less than one. Chi-squared test results may be invalid. ^1^Tests were not done because the column proportions are equal to one or zero SD: standard deviation, df: degrees of freedom

	Residency director				Fellowship director			
	Academic		Community		Academic		Community	
	Mean	SD	Mean	SD	Mean	SD	Mean	SD
Current focus	79.4^a^	14.8	70.7^a^	22.0	94.0^1^		^1^	
Ideal focus	65.7^a^	13.3	62.2^a^	15.6	57.8^a^	12.7	60.0^a^	6.0
Faculty and resource commitment	2.0^a^	1.3	3.1^b^	1.4	1.6^a^	1.0	2.6^a^	1.8
Faculty are satisfied	3.2^a^	1.0	3.7^b^	0.9	3.4^a^	0.9	3.8^a^	0.8

Tests of between-subjects effects							
Source			Type III sum of squares	df	Mean square	F-value	p-value
Director type	Faculty and resource commitment	4.135		1	4.135	2.660	0.106
	Faculty are satisfied	0.970		1	0.970	1.054	0.307
Institution type	Faculty and resource commitment	17.974		1	17.974	11.562	0.001
	Faculty are satisfied	3.756		1	3.756	4.079	0.046

## Discussion

We evaluated the research requirements, components, and general perceptions reported by 118 orthopedic surgery program and fellowship directors at ACGME-accredited academic and community training programs. The differences between academic and community programs, such as the level of concern over faculty and resource availability and the proportion of programs that were privately or publicly funded, were expected and consistent with previously reported data [5]. On the other hand, we did not anticipate several variables that were shared among all program directors regardless of their program designation, in particular, the number of programs that do not allocate protected time for research despite requiring it for graduation, the variability of the protected time when provided, and the shared desire to shift the focus of resident research toward basic science. These variables are actionable and impactful and warrant further discussion.

Time is perhaps the most invaluable yet overlooked resource to support research [5,6]. Despite a consensus on the importance of research during training and a near consensus (97.5%) of research activity as a graduation requirement, 45% of programs do not provide protected time to participate in research; those that do provide a median of nine weeks and six weeks at academic and community programs, respectively. In a study of 125 orthopedic residency programs, Williams et al. found that the type of dedicated research time correlates with residents’ research productivity [5]. Products such as posters and abstracts for local or institutional presentations were deemed unacceptable for the fulfillment of research requirements at 85% of the academic programs in our survey. These programs stipulated research as publishable manuscripts, clinical trial design and IRB submission, and regional or national podium presentations, all of which are considerably more time-intensive. ACGME guidelines for sponsoring institutions state that educational resources to support resident research should be provided [7]. Although academic programs provide research materials and dedicated personnel, these do little for residents if adequate time to access those resources is not also provided [8].

We also found an overall desire among directors to shift the focus of research toward basic science. This finding is consistent with reported trends. Current orthopedic literature shows waning numbers of preclinical publications and a concomitant increase in clinical publications without consistent increases in high-level evidence studies across subspecialties [9-12]. Furthermore, orthopedics has produced trailing rates of funded surgeon scientists and research dollars in the past decade compared to other surgical specialties [13]. Difficulty replenishing faculty members that are actively involved in research raises additional concerns about the present value and purpose of research in orthopedics [14]. Productive medical students and residents are more likely to match to a top-tier residency and fellowship program, respectively, and increasing numbers of students and trainees are participating in a research year [15]. There is no doubt that productivity in research is evidence of motivation; whether that motivation is genuine curiosity or to remain competitive in one of the most medical specialties is not known.

There are a number of limitations to our study. Our reported response rates were higher in our academic group than our community group, likely because this group is a self-selected group interested in research and supporting academic pursuits. Additionally, this cross-sectional study does not measure outcomes over time. Instead, our design allows for a larger subject inclusion for similar resources. Finally, while our survey was developed using published procedures for item development and study using a handbook for survey research in medical education [16], many questions are subjective measures and self-reporting standards that are subject to reporter biases. We also did not have access to individual resident data to validate some of the program/fellowship directors’ attitudes. For example, we had individual program directors who reported that >50% of their trainees were not completing their research requirements prior to graduation, yet there are no reports of programs with a less than 50% graduation rate.

The disproportionate productivity in clinical studies only, whether quality or not, is also understandable. Preclinical studies are time-consuming and expensive compared to clinical studies that can readily source data from an institutional medical record or insurance database. Nevertheless, preclinical studies are foundational for future advancements in orthopedics. In the last five years, 75% of the grants awarded to the field of orthopedics by the National Institute of Health went to a nonclinical (PhD) investigator, and at least half of the grants were directed toward basic science. The marked agreement among program directors regarding the ideal focus of orthopedic research directly aligns with the apparent interests of the single largest source of healthcare funding in the US. Given the responsibility of directors to graduate the next generation of orthopedic surgeons, their concerns should not be ignored.

## Conclusions

Despite the apparent differences, orthopedic surgery program directors at academic and community programs alike agree that a greater emphasis on basic science research is needed. Academic programs have historically championed basic science research. With fewer concerns over resources and funding, these programs must allocate significantly more time for research if a resurgence in surgeon scientists and preclinical discovery is to be achieved.

## Figures and Tables

**Figure 4:**
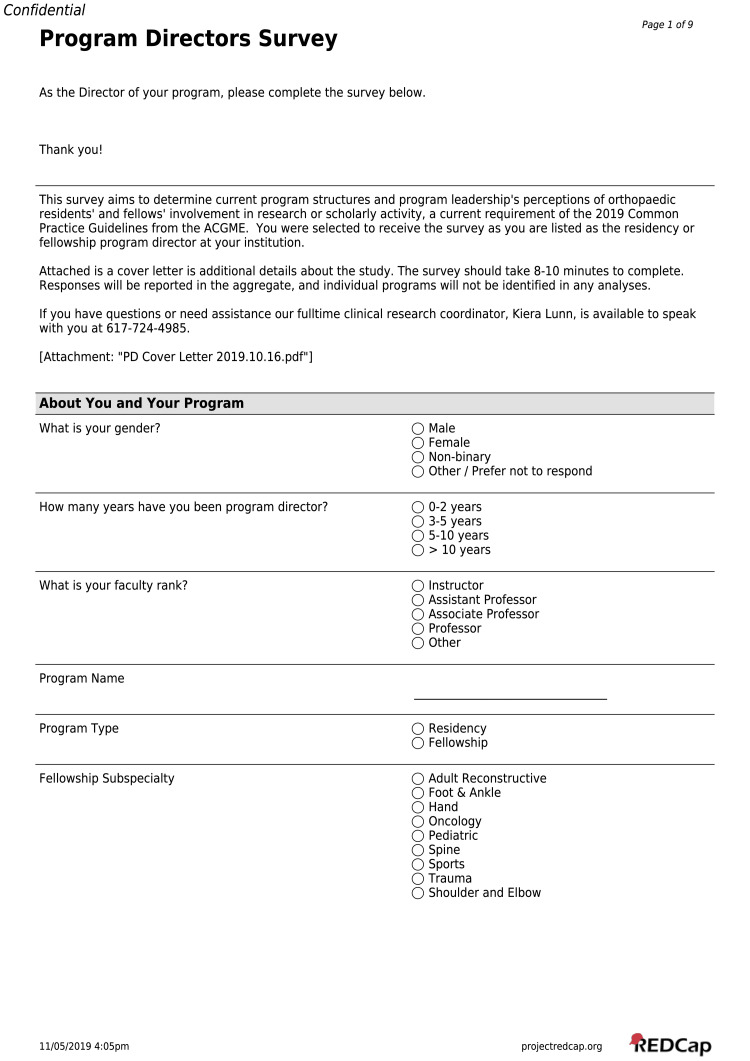
Program/Fellowship Director Survey, page 1

**Figure 5:**
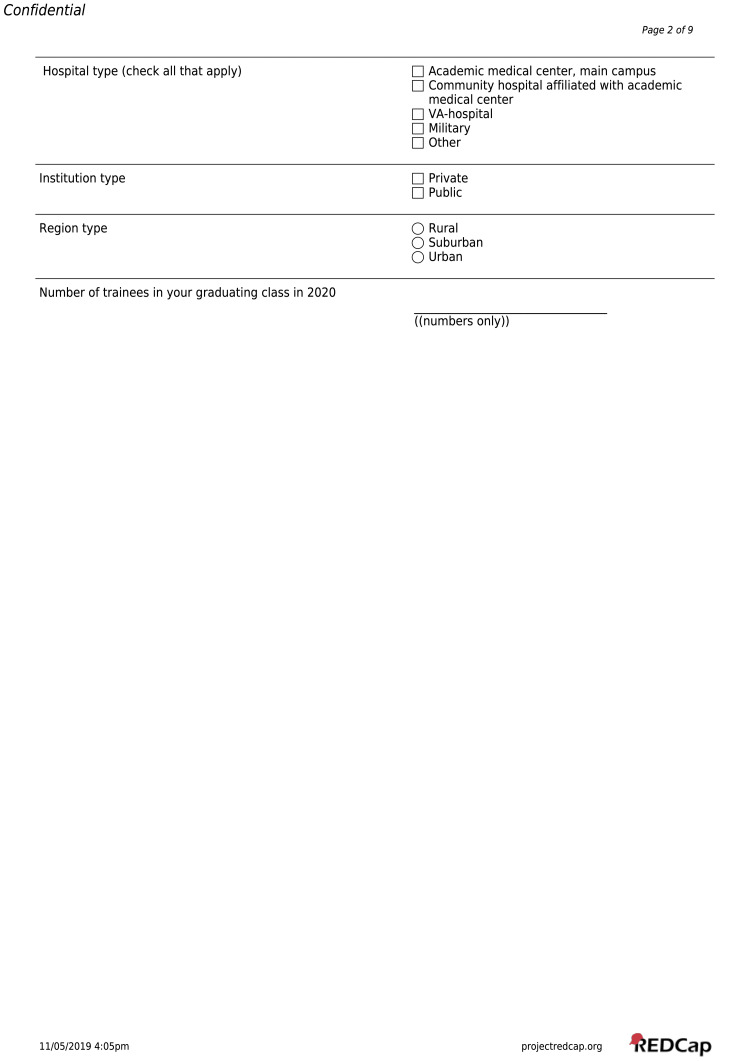
Program/Fellowship Director Survey, page 2

**Figure 6:**
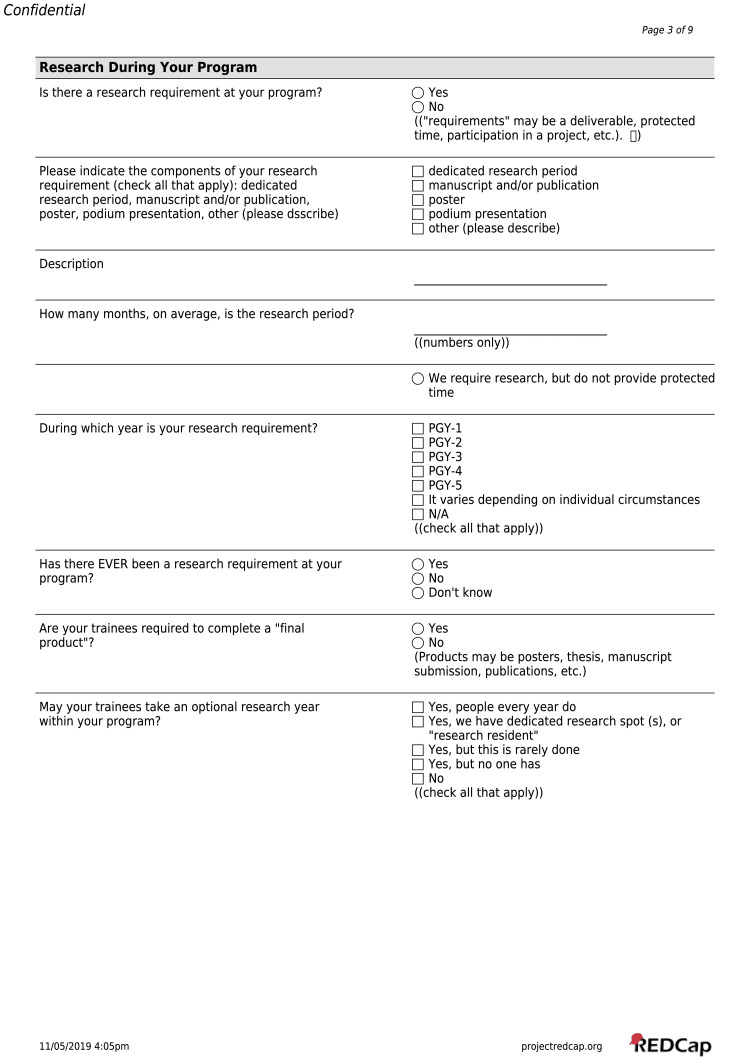
Program/Fellowship Director Survey, page 3

**Figure 7:**
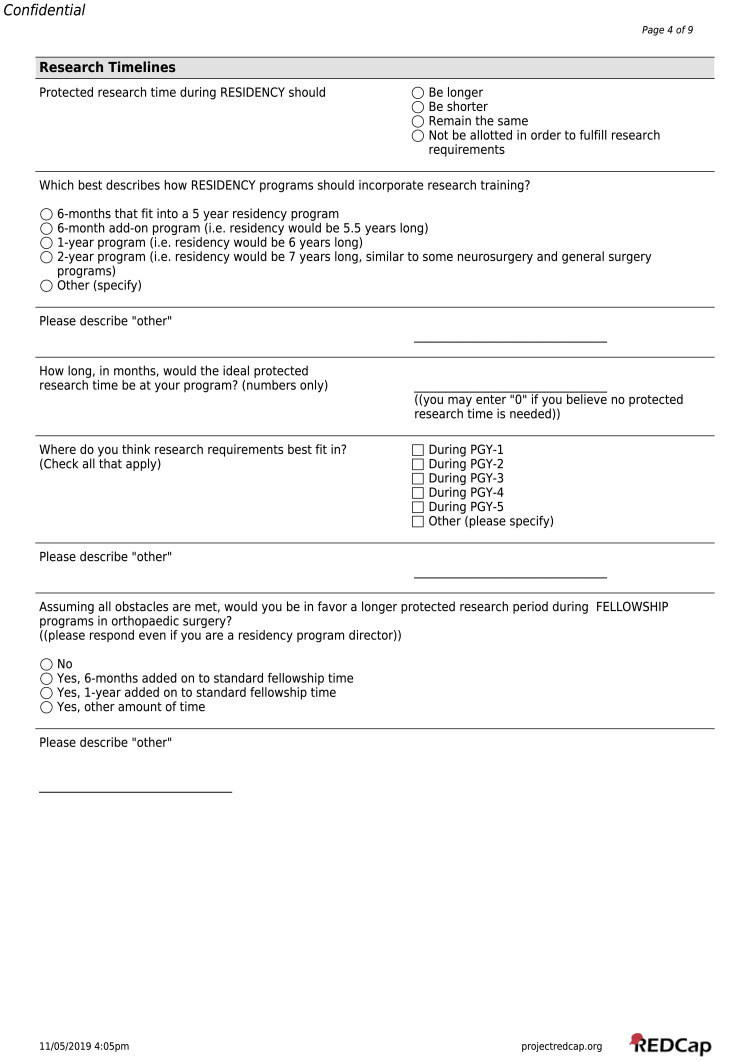
Program/Fellowship Director Survey, page 4

**Figure 8:**
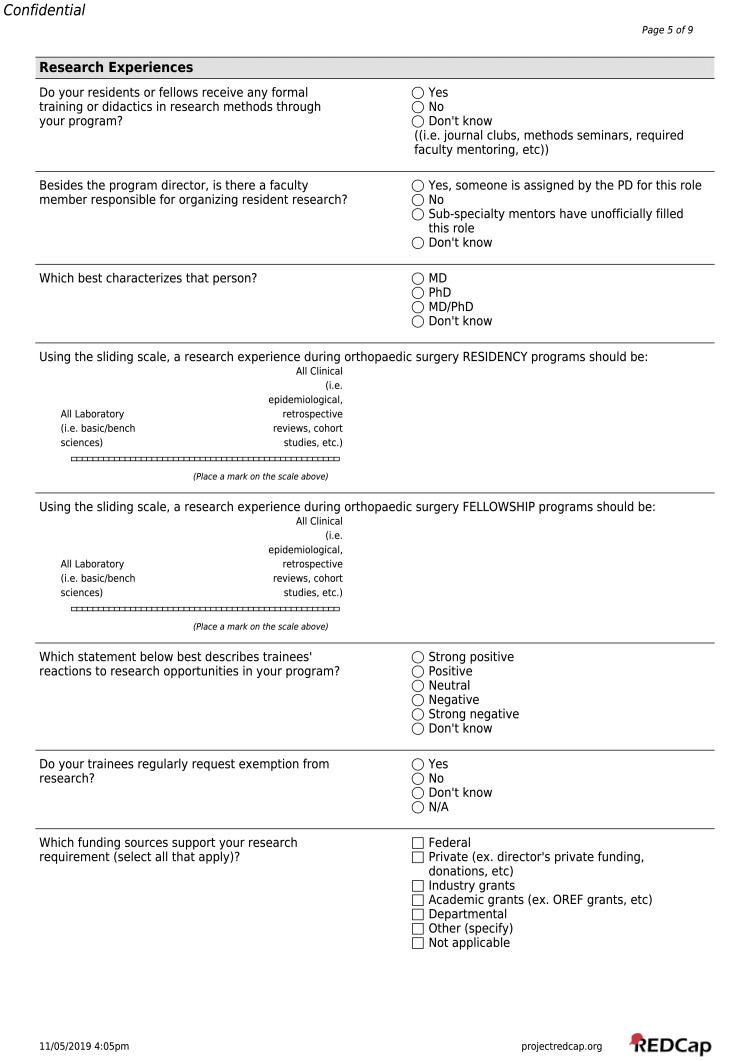
Program/Fellowship Director Survey, page 5

**Figure 9:**
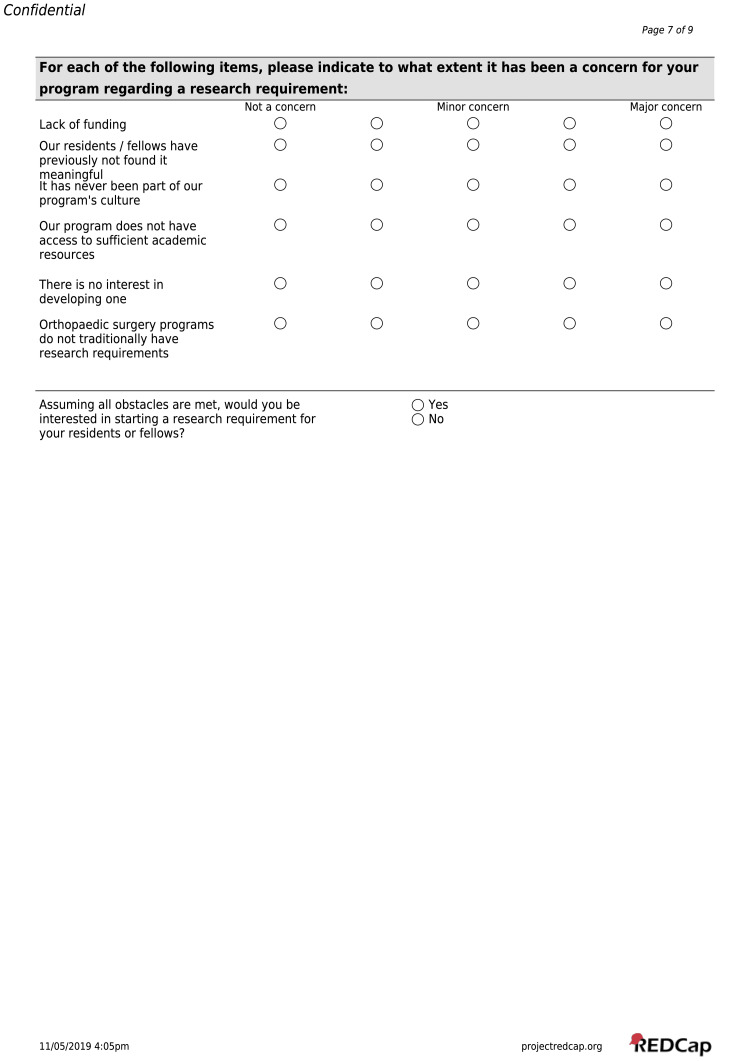
Program/Fellowship Director Survey, page 6

**Figure 10:**
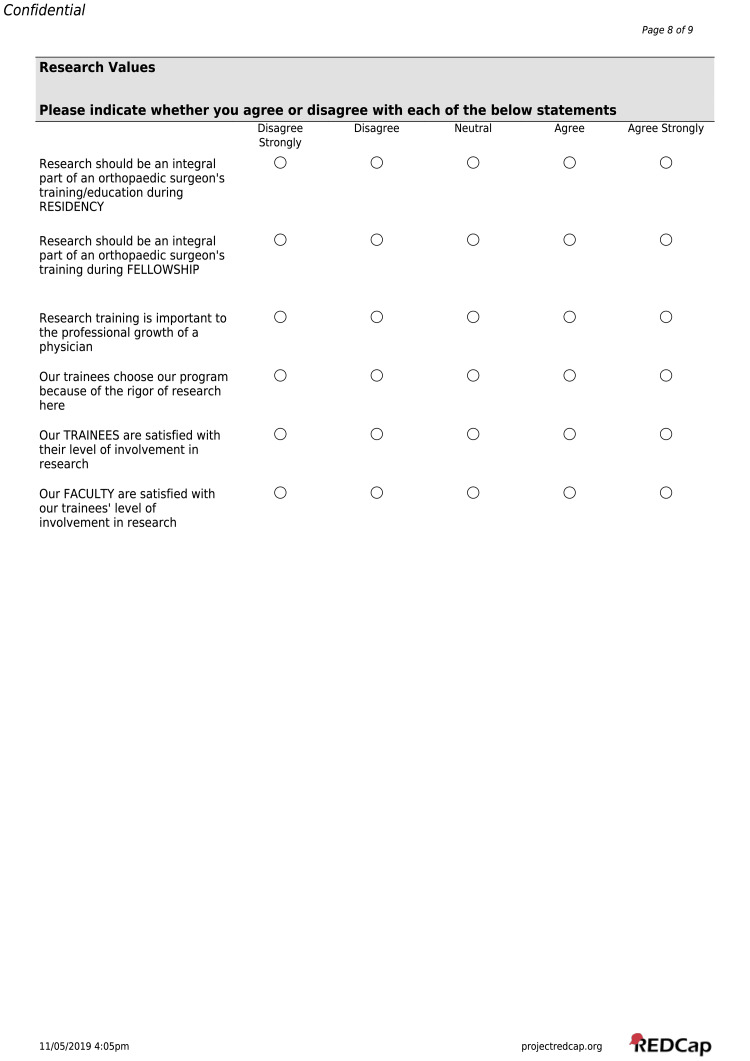
Program/Fellowship Director Survey, page 7

**Figure 11:**
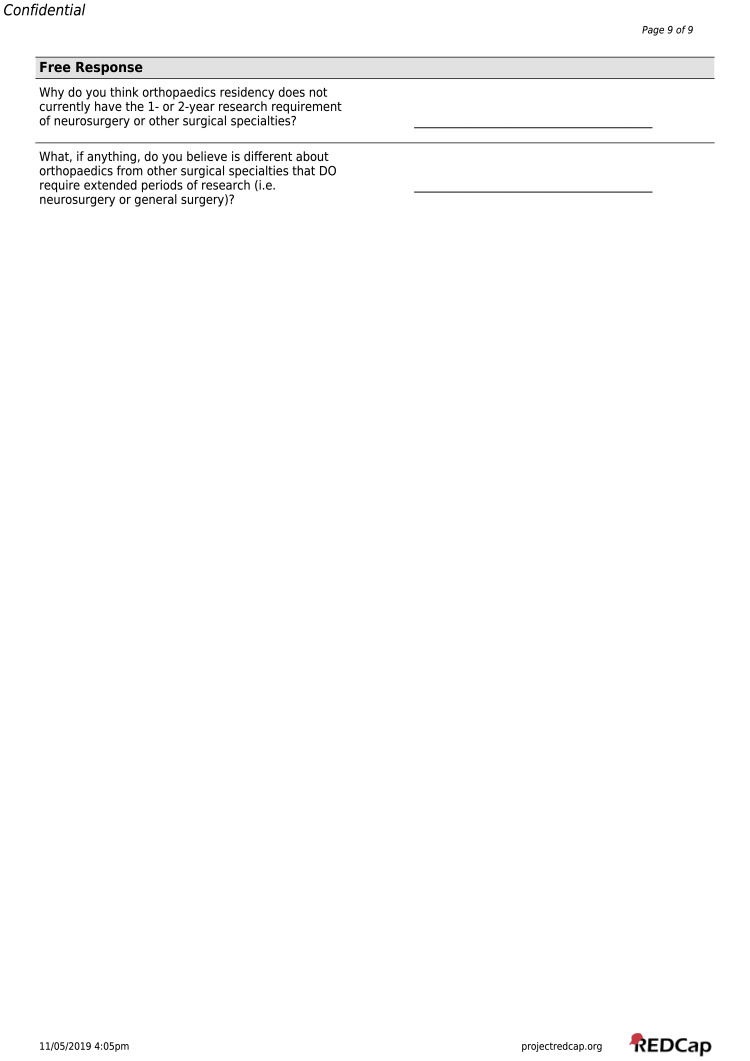
Program/Fellowship Director Survey, page 8

## Appendices

Program Directors Survey

As the Director of your program, please complete the survey below.

Thank you!

This survey aims to determine current program structures and program leadership’s perceptions of orthopedic residents’ and fellows’ involvement in research or scholarly activity, a current requirement of the 2019 Common Practice Guidelines from the ACGME. You were selected to receive the survey as you are listed as the residency or fellowship program director at your institution.

Attached is a cover letter with additional details about the study (Figures 4-11). The survey should take 8-10 minutes to complete. Responses will be reported in the aggregate, and individual programs will not be identified in any analyses.
